# Infant sleep state coded from respiration and its relationship to the developing functional connectome: A feasibility study

**DOI:** 10.1016/j.dcn.2025.101525

**Published:** 2025-02-09

**Authors:** Isabelle Mueller, Raimundo X. Rodriguez, Nicolò Pini, Cristin M. Holland, Rachel Ababio, Sanjana Inala, Kayla Delapenha, Venus Mahmoodi, Milana Khaitova, Xuejun Hao, William P. Fifer, Dustin Scheinost, Marisa N. Spann

**Affiliations:** a Department of Psychiatry, Vagelos College of Physicians and Surgeons, Columbia University, New York, NY 10032, United States; b Interdepartmental Neuroscience Program, Yale University, New Haven, CT 06520, United States; c Departments of Radiology and Biomedical Imaging, Yale School of Medicine, New Haven, CT 06520, United States; d Department of Pediatrics, Vagelos College of Physicians and Surgeons, Columbia University, New York, NY 10032, United States; e New York State Psychiatric Institute, New York, NY 10032, United States; f Child Study Center, Yale School of Medicine, New Haven, CT 06520, United States; g Department of Biomedical Engineering, Yale School of Engineering and Applied Science, New Haven, CT 06520, United States; h Department of Statistics and Data Science, Yale University, New Haven, CT 06511, United States; i Wu Tsai Institute, Yale University, New Haven, CT 06506, United States

**Keywords:** Active and quiet sleep, Connectomics, Sleep physiology, Functional magnetic resonance imaging

## Abstract

Most infants are scanned during natural sleep to maximize successful data acquisition by minimizing head and body motion. However, our understanding of how different sleep states affect the infant's functional connectome remains to be determined. In this feasibility study, we develop a novel approach to quantify active and quiet sleep during fMRI using time-locked infant respiration in twenty infants scanned within 47 weeks postmenstrual age. Sleep state (active versus quiet sleep) was then coded using established validated procedures from respiratory variability. Based on this sleep state coding, we investigated differences in the functional connectome comparing active versus quiet sleep. Eleven infants had sufficient quality respiration data to identify sleep states. There were no significant differences in the functional connectome of infants during active and quiet sleep. Still, large effect sizes existed, suggesting that sleep effects may be important in some studies. These findings demonstrate the feasibility and practicality of acquiring respiration data during scanning to facilitate sleep state coding and further understand its relationship to the neurodevelopment of infants. Given the relative ease of collecting respiration data using this setup, we conservatively recommend a wider adoption of our approach.

## Highlights

• We used a novel approach to quantify active and quiet sleep during fMRI using time-locked infant respiration.
• During scanning, we observed three times more active sleep than quiet sleep.
• Acquiring respiration data during scanning is a feasible and practical approach to derive sleep states.

## 1 Introduction

Functional magnetic resonance imaging (fMRI) has led to groundbreaking insights about the brain’s functional organization in early infancy (Pollatou et al., 2022). These results are particularly relevant for the infant functional connectome—a comprehensive, whole-brain map of functional connectivity (Craddock et al., 2013). The functional connectome rapidly develops over the first year (Damaraju et al., 2014, Li et al., 2024) and is a sensitive measure of neurodevelopmental risk and resilience (Allievi et al., 2016, Spann and Rogers, 2023, Yu et al., 2022). However, several challenges for infant connectomics still exist, including our understanding of how sleep states affect fMRI signals in infants (Korom et al., 2021). In contrast to older individuals, most infants are scanned during natural sleep to maximize the successful acquisition of data by minimizing head and body motion (Ellis et al., 2020, Korom et al., 2021). The impact of sleep on infant fMRI results remains unclear.

As sleep changes fMRI signals in older individuals (Larson-Prior et al., 2009, Tagliazucchi and Laufs, 2014, Tagliazucchi et al., 2013), understanding how natural sleep affects the infant's functional connectome is paramount for correctly interpreting infant fMRI results. For instance, functional connectomes in asleep 6- and 12-months-old infants more closely resemble functional connectomes in asleep adults than awake adults (Mitra et al., 2017). Similar results were found when comparing awake infants and adults during a naturalistic viewing paradigm (i.e., movie). The observed changes were driven mainly by frontoparietal regions (Yates et al., 2023). Together, these limited findings suggest some evidence of sleep effects found in infant fMRI results.

Nevertheless, sleep is not a single monolithic state. Young infants spend most of their time asleep, cycling through two distinct sleep states (Middlemiss, 2004). Active sleep is associated with low muscle tone, rapid eye movements (REM), and irregular breathing. In contrast, quiet sleep is defined by fewer movements, the absence of REM, and highly rhythmic respiration (Blumberg et al., 2020, Ktonas et al., 1990, Niemarkt et al., 2008, Scher et al., 2008, Werth et al., 2017). These two sleep states largely resemble REM sleep and non-REM sleep in adults and can be reliably coded from respiratory signals (Isler et al., 2016).

Electroencephalogram (EEG) studies have extensively demonstrated that cortical functioning exhibits significant differences between active sleep and quiet sleep across several EEG-derived parameters. These differences are particularly evident in measures of EEG functional connectivity (Tokariev et al., 2019, Tokariev et al., 2016, Yrjola et al., 2024), including phase-phase correlations, amplitude-amplitude correlations, and phase-amplitude correlations. Notably, these connectivity differences are frequency-specific and track the physiological maturation of structural cortico-cortical connectivity patterns. Active sleep is predominantly associated with desynchronized cortical activity, facilitating dynamic neural interactions and possibly supporting sensorimotor and cognitive processing; quiet sleep is characterized by synchronized slow-wave activity, which may play a critical role in processes like synaptic consolidation and restorative neural functions (Tokariev et al., 2019, Tokariev et al., 2016, Yrjola et al., 2024).

Adding to these findings, converging evidence from multiple neuroimaging modalities, such as near-infrared spectroscopy (fNIRS), provides a broader perspective on these state-dependent connectivity patterns (Lee et al., 2020, Uchitel et al., 2023, Vanhatalo et al., 2002). Specifically, active sleep is linked to enhanced interhemispheric connectivity, indicating robust communication across hemispheres, while quiet sleep tends to exhibit stronger short-range functional connectivity, emphasizing localized neural processing (Lee et al., 2020, Uchitel et al., 2023, Vanhatalo et al., 2002). Together, these complementary evidence underscores the distinct neural dynamics underlying active and quiet sleep, highlighting that multiple sleep states—rather than a binary awake vs asleep classification—may need to be considered when interpreting functional neuroimaging data. No studies have investigated how sleep states (active vs quiet sleep) affect an infant’s functional connectome when measured with fMRI.

In this feasibility study, we develop a novel approach to measure respiratory variability during fMRI scanning for coding infant sleep states. Respiratory signals—time-locked with fMRI acquisition—were collected via a pressure pad placed under the sleeping infant. The sleep state (active vs quiet sleep) was then coded using established, validated procedures based on respiratory variability (Isler et al., 2016). Based on this sleep coding, we investigated differences in the functional connectome between active and quiet sleep in eleven infants under two months using fMRI.

## 2 Material and methods

### 2.1 Participants

The sample for this study was drawn from larger longitudinal studies aimed at understanding prenatal factors on offspring neurodevelopment across pregnancy through infancy. For the studies, participants were recruited through the Department of Obstetrics and Gynecology at Columbia University Medical Center and advertisement. Inclusion for enrollment included a healthy singleton pregnancy course, routine prenatal care, and being primarily English-speaking at the time of enrollment. Exclusion criteria included psychotropic medications, smoking, drug use, or heavy alcohol use, and multifetal pregnancy. After birth, offspring were included if they were born ≥ 37 weeks, had a birthweight of more than 2500 g, and had no major prenatal or delivery complications. Exclusion criteria for infants included congenital anomalies, ferromagnetic implants, and admission to the neonatal intensive care unit for longer than 24 hours. The Institutional Review Board of the New York State Psychiatric Institute approved all study procedures. Written informed consent was obtained from participants in the study.

Of the initial twenty infants, seven infants were excluded due to insufficient quality of the respiratory signal. Two infants were excluded due to errors in time locking the fMRI and respiratory signal. The final sample of eleven infants (female=6; 45.5 %) had a mean postmenstrual age of 45.9 weeks at the MRI scan. Most infants were Hispanic ethnicity (63.6 %) as Hispanic/Latino by parent report.

### 2.2 Infant imaging

**Fig. 1:**
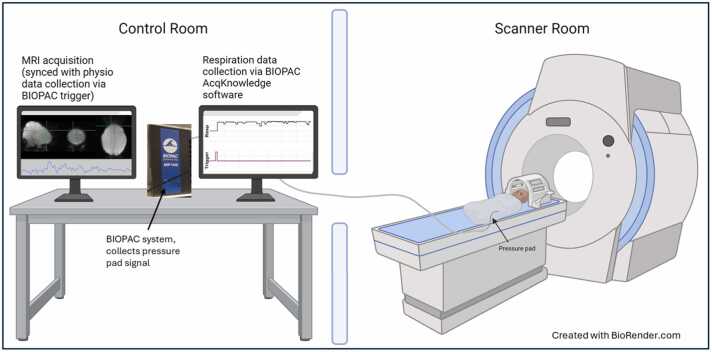
Schematic of scanning setup. During the preparation of the infant with standard procedure (Korom et al., 2021), the pressure pad is taped to the infant's abdomen. Once in the scanner, the pressure pad is connected to a BIOPAC system. The BIOPAC system records the respiratory signal from the pressure pad and triggers from the functional sequence. During the functional runs, the respiratory signals—time-locked with fMRI acquisition—were coded to derive sleep state assignment (active versus quiet).

Twenty infants were scanned within the first weeks of postmenstrual life (PMA ≤ 46 weeks). After they were fed and swaddled, they were given time to fall asleep naturally while listening to a recording of the scanner sounds from each pulse sequence to acclimate to the scanning environment. Foam ear plugs, wax, and ear shields (Natus Medical) were used to dampen the scanner noise. Respiration was derived using a pressure pad (BIOPAC TSD110-MRI; Biopac Systems, Inc) taped to the infant’s lower rib cage at the midclavicular line (Fig. 1). The respiration signals were sampled with a frequency of 500 Hz using a BIOPAC MP150 data acquisition system and a DA100C amplifier.

MRI data were acquired using a 3 Tesla General Electric Signa Premier with a 48-channel head coil. The anatomical T2-weighted images were acquired with: TR = 3202 ms; TE = 60 ms; matrix size = 256 × 256; FOV = 256 × 256 mm; phase FOV = 100 %; ETL = 140; slice thickness = 0.9 mm. Functional images were acquired using a standard echoplanar imaging sequence: TR = 2000 ms; TE = 30 ms; matrix size = 64 × 64; FOV = 190 × 190 mm; phase FOV = 100 %; slice thickness = 3.0 mm; number of slices = 34; bandwidth = 7812.5 Hz; voxel size = 2.969 × 2.969 × 3 mm; number of volumes = 90. The functional sequences had built-in discarded volumes to allow the tissue to reach a steady state. The number of runs varied per participant due to participant compliance (e.g., infant waking and moving). In total, fifty runs were collected for a median of five runs per infant.

### 2.3 Image processing

Anatomical images were skull-stripped using FSL. If, after visual inspection, any non-brain tissue remained, it was removed manually. Unless otherwise specified, all further analyses were performed using BioImage Suite (Joshi et al., 2011). Anatomical images were non-linearly registered to a custom, age-appropriate template (Spann et al., 2018) using a validated algorithm (Scheinost et al., 2017). After the anatomical scans were registered to the template, functional images were rigidly aligned to the anatomical images. All transformation pairs were calculated independently and combined into a single transform. This single transformation allows common space images to be transformed to the individual participant with only one transformation, thereby reducing interpolation error.

Data were cleaned as previously described (Scheinost et al., 2020). We performed motion correction on the functional data with SPM12. The frame-to-frame motion was calculated across all the functional volumes. Linear and quadratic drifts, mean cerebrospinal fluid signal, mean white matter signal, mean gray matter signal, and a 24-parameter motion model (6 motion parameters, six temporal derivatives, and their squares) were regressed from the data. A Gaussian filter with an approximate cutoff frequency of 0.12 Hz was used to smooth the functional data temporarily. Because motion affects functional connectivity measures (Van Dijk et al., 2012), we used a strict inclusion criterion of an average frame-to-frame motion < 0.15 mm.

An infant-specific parcellation was used (Scheinost et al., 2016). First, the atlas was warped into single-subject space using the transformation described above. Next, the mean time series from each functionally defined node was calculated by averaging its constituent voxels' time series data. Pairwise correlations were computed between all node-pair combinations. These correlations were Fisher z-transformed to produce symmetric 89 × 89 connectomes.

### 2.4 Respiratory signal processing and sleep state coding

**Fig. 2:**
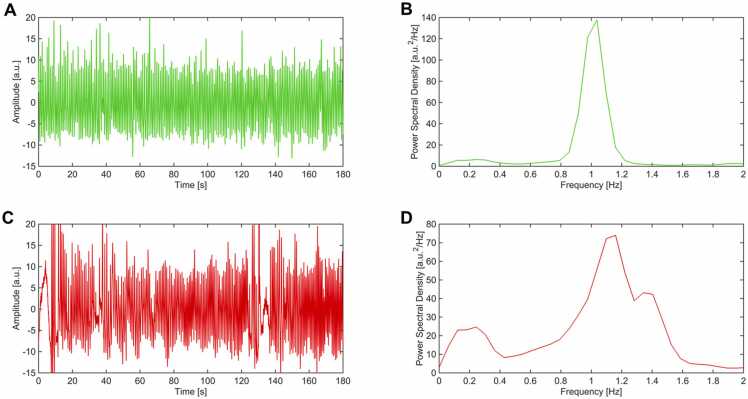
Exemplar respiratory traces and power spectral densities from consecutive functional runs for a single infant. A) Respiratory traces in quiet sleep have stable amplitude and interbreath variability. B) Accordingly, the corresponding power spectral density has a narrow, isolated frequency peak centered around 1 Hz. C) Respiratory traces in active sleep have large amplitude and interbreath variations. D) Accordingly, the corresponding power spectral density multiple frequency peaks over a broad range of frequencies.

We used a validated approach to code infant sleep (Isler et al., 2016). This approach has a better than 80 % concordance with standard polysomnographic state coding. In infants, the average breathing frequency range is between 20 and 80 breaths per minute (bpm), corresponding to a respiratory frequency of 0.3 Hz and 1.3 Hz, respectively. The respiration signals were band-pass filtered (using low-pass and high-pass infinite impulse response filters) in the frequency range 0.05–5 Hz (Lucchini et al., 2018, Pini et al., 2019). Power spectral densities were obtained using the Welch method, considering a window of width 10 seconds and 50 % overlap between adjacent windows. Infant sleep states were coded as active sleep, quiet sleep, or indeterminate based on a combination of the morphological characteristics of the respiratory signals and frequency content of the power spectral densities for instantaneous breathing rate using Gmark (Ledano Solutions) (Isler et al., 2016). Specifically, active sleep, compared to quiet sleep, is characterized by a substantially higher breathing frequency and higher variability in the instantaneous breathing rate (Fig. 2). Accordingly, power spectral densities of the respiratory signals for quiet sleep are narrow and centered at a signal frequency, whereas, for active sleep, they are broad with multiple peaks. Subsequent analysis included segments coded as active or quiet sleep. Indeterminate segments were excluded. The minimum active or quiet sleep state duration was set to 3 consecutive minutes (i.e., a whole functional scan) (Prechtl, 1974).

### 2.5 Statistical analyses

We used edgewise linear mixed-effects models to compare functional connectivity between active and quiet sleep. Linear mixed-effect models allow participants to have variable numbers of active and quiet sleep scans and were implemented using AFNI's 3dLME function (Chen et al., 2013). Each edge of the connectome was the dependent variable. Sleep state was the independent variable. Participant was a random intercept. Models adjusting for postmenstrual age at scan, sex, and mean head motion were also performed. The resulting p-values were corrected for false discovery rate (FDR) using the Benjamini-Yekutieli procedure (Yoav and Daniel, 2001). Given that significance is a poor marker of an effect’s meaningfulness, we also assessed the effect size of the association between sleep state and functional connectome. We used the lme.dscore function in R to convert the test statistics (i.e., t-values) to effect sizes—measured as Cohen's D. Effects were labeled as small (0.2 ≤|D|<0.5), medium (0.5 ≤|D|<0.8), and large (|D|≥0.8) based on standard recommendations (Sullivan and Feinn, 2012).

## 3 Results

### 3.1 Infant sleep coding

**Fig. 3:**
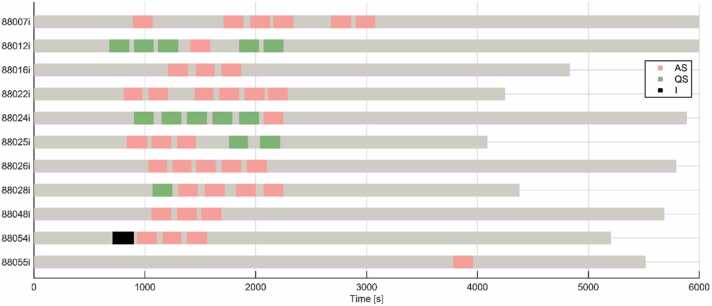
Temporal progression of active (AS) and quiet sleep (QS) states over a scan session. The number and start times of functional runs varied during scanning based on infant compliance. For most infants, functional runs began around 15 minutes into the scanning session, and a median of 5 runs was acquired per infant. A majority of runs were in AS, and only 4 infants went into QS during a functional run. Pink blocks represent functional runs in AS. Green blocks represent functional runs in QS. Black blocks represent functional runs with indeterminate sleep state. Grey blocks represent non-functional sequences or time in the scanner where sleep was not coded.

Sleep state was coded successfully for 49 out of 50 (98 %) functional runs, of which a majority were coded as active sleep (active sleep: 36 runs; quiet sleep: 13 runs; $\chi^{2}$=6.67 p = 0.01). Only four infants entered quiet sleep, while all infants entered active sleep. Five runs coded as active sleep and two runs coded as quiet sleep were removed for motion greater than 0.15 mm. Head motion was not significantly different between active and quiet sleep (t = 1.69, p = 0.1; active sleep: 0.049 mm, quiet sleep: 0.029 mm). Fig. 3 shows how infants progress through sleeps states over the course of a scan.

**Fig. 4:**
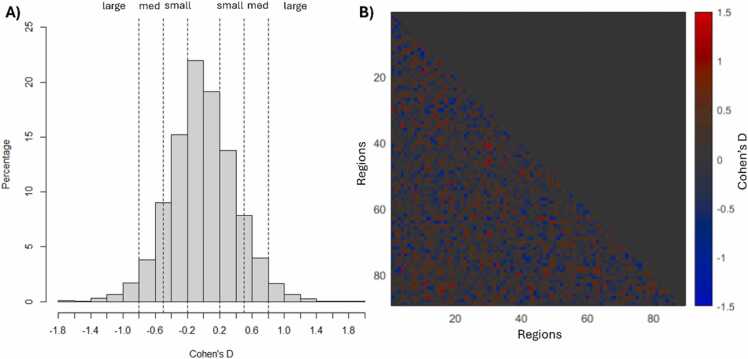
Effect sizes for comparing active and quiet sleep. A) Most edges showed minimal effect sizes with ∼45 % exhibiting |D|< 0.2. Only 3.5 % of edges had large effect sizes(|D|≥0.8). B) Spatial distribution of effect sizes.

### 3.2 Associations between sleep state and functional connectome

No significant edges were observed after False-Discovery Rate (FDR) correction. Models adjusting for age, sex, and mean head motion were also not significant. When an unadjusted model was used, 3.5 % of edges had a large effect size (|D|≥0.8), 14.6 % of edges had a medium effect size (0.5 ≤|D|<0.8), and 36.7 % of edges had a small effect size (0.2 ≤|D|<0.5). The average effect size for edges greater in active sleep was Cohen’s D= 0.30; the average effect size for edges greater in quiet sleep was Cohen’s D= 0.30. The distribution of effect sizes (shown in Fig. 3a) was roughly Gaussian and symmetric around 0, suggesting that any connectivity changes associated with sleep state are not in a single direction.

### 3.3 Post-hoc power analysis

We performed a post-hoc power analysis to facilitate future studies on significance testing between quiet and active sleep. If we restrict our analyses to the high effect size edges and assume each has Cohen’s D of 0.8, a total sample of 137 infants would be needed using the Bonferroni correction for multiple comparisons. Since the Bonferroni correction is overly conservative, fewer participants may be needed when using other multiple comparison methods, such as FDR or cluster correction.

## 4 Discussion

This study demonstrated the feasibility of a novel approach to quantify active and quiet sleep during fMRI using time-locked infant respiration. Findings indicate that sufficient quality respiration data can be obtained using a pressure pad and standard physiological recording equipment to identify sleep states. During scanning, infants were more likely to enter active sleep than quiet sleep periods. No significant differences in the functional connectome were observed during active and quiet sleep in our sample. Still, large effect sizes existed, suggesting connectivity differences in specific edges between active and quiet sleep may be observable in future studies with larger sample sizes. As this area of investigation is novel, these findings demonstrate the feasibility and practicality of acquiring respiration data to facilitate sleep state coding and further understand neurodevelopment in infants.

Beyond infancy, few studies have compared functional connectivity between REM and non-REM sleep. Most have highlighted default-mode network connectivity changes with sleep state (Horovitz et al., 2009, Houldin et al., 2021, Sämann et al., 2011). Nevertheless, some have reported no changes in the default-mode network between REM and non-REM sleep (Koike et al., 2011). In infants, activity differences between active and quiet sleep using task-based functional near-infrared spectroscopy (fNIRS) are inconsistent (Kotilahti et al., 2005, Kotilahti et al., 2010). Like ours, these studies often only included a few participants scanned in each sleep state. For example, six of thirty-four participants entered REM sleep (Houldin et al., 2021) in one study. Other successfully scanned < 10 participants in both sleep states (non-REM and REM) (Braun et al., 1998, Koike et al., 2011). These sample sizes are consistent with our study. The results suggest that collecting multiple states during natural sleep is difficult in the scanner. When participants fall asleep, how they cycle between different sleep states are beyond the experimenter’s control.

Infants at this age spend ∼40 % of their sleep time in quiet sleep (Middlemiss, 2004). However, despite multiple functional runs, only four entered quiet sleep in our study. There may be several reasons for the lack of QS. First, newborns sleep onset occurs through AS, not QS. Second, the scanning environment may bias infants towards a particular sleep state. For example, fMRI sequences are loud, with high-pitched buzzing and repetitive knocking sounds, which may prevent some infants from entering deeper sleep. Third, we only coded the functional runs for sleep. Infants may have entered QS during structural sequences. Much larger sample sizes, such as the HEALthy Brain and Child Development (HBCD) Study (Dean et al., 2024, Nelson et al., 2024, Volkow et al., 2024), will be needed to fully understand how MRI scanning influences which sleep states are observed.

The need to account for sleep state in infant fMRI is unclear. On one hand, differences between active and quiet sleep were insignificant and 80 % of edges exhibited a small or very small effect size of sleep state. Also, quiet sleep was uncommon. Only 26 % of the 150 minutes of fMRI data was coded as quiet sleep, and four out of eleven infants entered quiet sleep. Finally, an early work reported that associations between connectivity and postmenstrual age were the same in naturally sleeping and sedated infants (Doria et al., 2010). Moreover, no differences in hemodynamic responses to speech and music using fNIRS were observed between quiet and awake sleep (Kotilahti et al., 2010). On the other hand, some high effect size edges exist, suggesting that sleep state could be an important variable for accurately interpreting results, at least in some regions. Given the relative ease of collecting time stamped respiration data using a pressure pad, a conservative consideration may be to collect these data. They can be used later to code for sleep or in advanced fMRI data cleaning methods if desired.

This study has several strengths. All data were acquired prospectively. The sample includes infants under two months of age and, thus, minimizes the possibilities of developmental changes in sleep patterns, which occur around six months (Ktonas et al., 1990). The sample was predominantly Hispanic/Latino, an underrepresented biomedical research population. While our sample size is small, we used 42 functional runs and (on average) nearly 11.5 minutes of data per participant in our analyses because of LME framework. This framework can leverage with-in participant comparisons for those with both quiet and active sleep. Such an approach increases our power despite our sample size. Additionally, we evaluated effect sizes instead of focusing on significance, which others can use to power comprehensive studies. Our setup for collecting respiratory data using a small animal pressure pad is relatively simple and inexpensive. Several vendors (i.e., BIOPAC) and scanner manufacturers (i.e., Siemens) sell this equipment. Using a pad avoids possible skin irritation from electrodes, increasing infant comfort. Additionally, our approach does not need video cameras during scanning to encode sleep states, increasing the practicality and feasibility and reducing privacy concerns of identifying video data.

This study has several limitations. Ongoing challenges exist with data quality for the physiologic data during the MRI scan. The respiratory data during scanning is generally noisy and even noisier during the fMRI sequences. Further algorithmic developments are needed to improve data quality and reduce data loss. We only observed functional runs with a single sleep state, likely due to the short duration of our runs (3 minutes). In the future, longer scan lengths may allow an infant to progress into QS and facilitate traditional time series analyses (i.e., GLMs). These analyses with physiologic and functional data may retain more sleep data. Since movement differences are part of the definitions of sleep state, head motion, and sleep state cannot be uncoupled. Still, head motion in both states was exceptionally low, and similar effect sizes were observed when controlling for head motion. We restricted our analyses to infants under two months. Infant sleep patterns change around three to six months (Ktonas et al., 1990, Middlemiss, 2004). Thus, results may not be the same in older infants. Sleep coding based on respiration has 80–90 % concordance with the gold standard, polysomnographic coding (Isler et al., 2016). Some differences in coded sleep state may exist with different coding schemes.

In conclusion, we demonstrate a novel method for collecting respiratory data using a pressure pad to code infant sleep during scanning. We provide initial validation and investigate how active and quiet sleep affect the infant's functional connectome. Overall, this novel, noninvasive methodology for infant physiology acquisition can support further investigation into active and quiet sleep states and the developing brain across infancy. Future studies should investigate associations among the infant functional connectome and sleep states in larger and more diverse samples.

## CRediT authorship contribution statement

**Hao Xuejun:** Writing – review & editing, Investigation, Formal analysis. **Fifer William P.:** Writing – review & editing, Validation, Supervision, Resources, Project administration, Investigation, Formal analysis, Data curation. **Scheinost Dustin:** Writing – review & editing, Writing – original draft, Visualization, Supervision, Project administration, Investigation, Formal analysis, Conceptualization. **Mueller Isabelle:** Writing – review & editing, Writing – original draft, Data curation. **Spann Marisa N.:** Writing – review & editing, Writing – original draft, Visualization, Validation, Supervision, Software, Resources, Project administration, Methodology, Investigation, Funding acquisition, Formal analysis, Data curation, Conceptualization. **Rodriguez Raimundo X.:** Writing – review & editing, Visualization, Formal analysis, Data curation. **Pini Nicolò:** Writing – review & editing, Visualization, Data curation. **Holland Cristin M:** Writing – review & editing, Data curation. **Ababio Rachel:** Writing – review & editing, Visualization, Data curation. **Inala Sanjana:** Writing – review & editing, Project administration, Data curation. **Delapenha Kayla:** Writing – review & editing, Visualization, Data curation. **Mahmoodi Venus:** Writing – review & editing, Project administration. **Kaitova Milana:** Writing – review & editing, Investigation, Data curation.

## Declaration of Competing Interest

The authors declare that they have no known competing financial interests or personal relationships that could have appeared to influence the work reported in this paper.

## Data Availability

The authors do not have permission to share data.
